# Coronary artery fistula with associated Takotsubo cardiomyopathy: a case report

**DOI:** 10.1186/s13256-018-1567-5

**Published:** 2018-03-30

**Authors:** Rabail Qureshi, Leo Kao, Rakesh P. Gupta

**Affiliations:** 1 Department of Internal Medicine, Flushing Hospital Medical Center, Flushing, NY USA; 2 Department of Interventional Cardiology, Flushing Hospital Medical Center, Flushing, NY USA

**Keywords:** Acute coronary syndrome, Angina pectoris, Coronary artery fistula, Microfistula, Takotsubo cardiomyopathy

## Abstract

**Background:**

Coronary artery fistula, first described by Krause in 1865, is an abnormal communication between the coronary artery and one of the four chambers of the heart or one of the great vessels. The communications are often congenital but may also be acquired from trauma or invasive cardiovascular procedures. Half of the cases present with angina pectoris whereas the remaining half are incidentally detected on echocardiogram or angiogram performed for an unrelated reason.

Takotsubo cardiomyopathy or stress-induced cardiomyopathy is characterized by transient left ventricular dysfunction with minimal elevation of cardiac biomarkers in the absence of underlying coronary artery disease. Almost 90% of reported patients are postmenopausal women with a history of recent emotional or physical stress.

**Case presentation:**

We report an unusual case of a 64-year-old Hispanic woman presenting with typical symptoms suggestive of acute coronary syndrome after an extreme familial conflict. There was mild troponin elevation. Cardiac catheterization revealed microfistulas originating from the third portion of the left anterior descending artery draining to the left ventricular cavity. The ventriculogram demonstrated the apical ballooning. We postulate that high local concentration of catecholamine triggered by stress resulted in angina pectoris due to worsening coronary steal from the coronary fistula. Also, the stress-induced adrenergic stimulation unmasked the classical akinetic apex and apical ballooning characteristic of Takotsubo cardiomyopathy.

**Conclusions:**

This case report highlights the rare but important association between two uncommon conditions. To the best of our knowledge, only one similar case has been reported describing a patient with microfistulas to left ventricular cavity and concurrent Takotsubo cardiomyopathy.

## Background

A coronary artery fistula (CAF) is an abnormal communication between the coronary artery and one of the four chambers of heart or one of the great vessels. These communications are most often congenital. Most of the cases arise from the right coronary artery (50–60%) and the left anterior descending (LAD) artery (25–42%); followed by the circumflex coronary, diagonal, and left main coronary artery (18.3%, 1.9%, and 0.7% respectively). The condition can also be acquired from trauma, congenital heart surgery, transcutaneous techniques used for myocardial biopsy, percutaneous coronary intervention, or as a sequela of Kawaski disease. The fistulous connection results in blood bypassing the myocardial capillary network and a coronary steal that manifests as acute coronary syndrome (ACS).

Takotsubo cardiomyopathy (TCM) is characterized by transient left ventricular dysfunction and minimal elevation of cardiac biomarkers in the absence of underlying coronary artery disease. First described in Japan in 1991, the condition is named Takotsubo due to the morphological association of the left ventricle to a Japanese octopus trapping pot consisting of a round bottom and narrow neck. It is commonly seen in women with a recent history of physical or emotional stress. Despite the dramatic initial presentation and compromised left ventricular systolic function almost all patients recover fully.

This case highlights rare association between the above two entities and only one such association is reported in past [1].

## Case presentation

A 64-year-old Hispanic woman with a past medical history of hypertension, hyperlipidemia, and transient ischemic attack presented with a chief complaint of chest pain. The pain was severe enough to wake her from sleep around 6.00 a.m. Pain was 8/10 and described as pressure and as if someone had punched her on left side of her chest with an associated tingling sensation in her left arm and fingers. She related her chest discomfort to the recent familial conflict. The pain did not improve over the next hour, and she proceeded to walk a few blocks to the hospital. A review of her symptoms was negative. Her medications included aspirin 81 mg and lopressor 25 mg. Our patient denied a history of coronary artery disease, tobacco smoking and alcoholism, a family history of coronary artery disease or invasive cardiovascular procedures. On arrival at 7.02 a.m., the first set of vitals showed a pulse rate of 79, a respiratory rate of 18, blood pressure of 166/87 and oxygen saturation 100% on 2 L via nasal cannula. A physical examination revealed a body mass index (BMI) of 25.3. She was anxious and was in obvious discomfort. The remainder of the examination was unremarkable. An electrocardiogram (EKG) showed normal sinus rhythm at 77 per minute with normal ST and T wave. A chest X-ray revealed a normal cardiac silhouette. Her baseline troponin was within normal limits. Lopressor, aspirin, and nitroglycerin were administered in the emergency department (ED). However, her pain worsened. The repeat troponin tests at 4-hourly interval trended up from < 0.012 baseline to 0.736 . The repeat EKG did not display any ischemic ST-T wave changes to explain troponin elevation or to account for the degree of pain. A Stat transthoracic echocardiogram (TTE) yielded normal left ventricular (LV) ejection fraction and normal wall motion. (The Stat TTE was performed due to the persistent pain being unresponsive to nitroglycerine and morphine.)

Our patient was managed with an ACS protocol with acetylsalicylic acid (ASA) 325 mg, plavix 600 mg, full-dose lovenox, lopressor 100 mg, lipitor 80 mg, and underwent emergent cardiac catheterization. On angiogram, no atherosclerotic or stenotic lesion was visualized; however, following the injection of contrast medium, a plexiform network of vessels in the LV wall was demonstrated, followed by a jet of contrast medium entering the left ventricular cavity originating from the distal third of the left anterior descending and diagonal arteries. The plexus was eventually identified as coronary artery microfistulae. All fistulae drained into the left ventricular cavity. The ventriculogram elicited apical ballooning of the left ventricle with preserved function of the remaining myocardium consistent with TCM.

The echocardiogram (ECHO) did not reveal the apical ballooning seen on the ventriculogram likely due to two reasons:

First, because of it was a technically difficult study; second, as the Takotsubo is a transient phenomenon, it is likely that she developed the apical ballooning while being transferred for the cardiac catheterization.

After medical treatment our patient was discharged on metoprolol and aspirin with full resolution of symptoms and remained asymptomatic at 1-month and 6-month follow-up.

## Discussion

According to the Ogden’s classification, the coronary artery fistula is an anomaly of termination representing an abnormal communication between the coronary arteries and the cardiac chambers [coronary-cameral fistulae (CCF)] or the low pressure veins (coronary arteriovenous malformations) [2]. The overall incidence is about 0.002. The most common etiology is congenital, representing about 0.4% of all congenital heart defects, other frequent causes include trauma and invasive cardiovascular procedures.

In congenital cases, the incidence of coronary artery fistulas is much higher for the right heart chambers as compared to left due to lower pressure with 45% cases involving the right ventricle, 25% right atrium and 15% affecting the pulmonary artery [3]. The left heart constitute about < 10% of the cases [4]. With right-sided fistulas, the hemodynamics resemble those of extracardiac left-to-right shunt and the left-sided fistulas usually mimic aortic insufficiency [5].

**Fig. 1:**
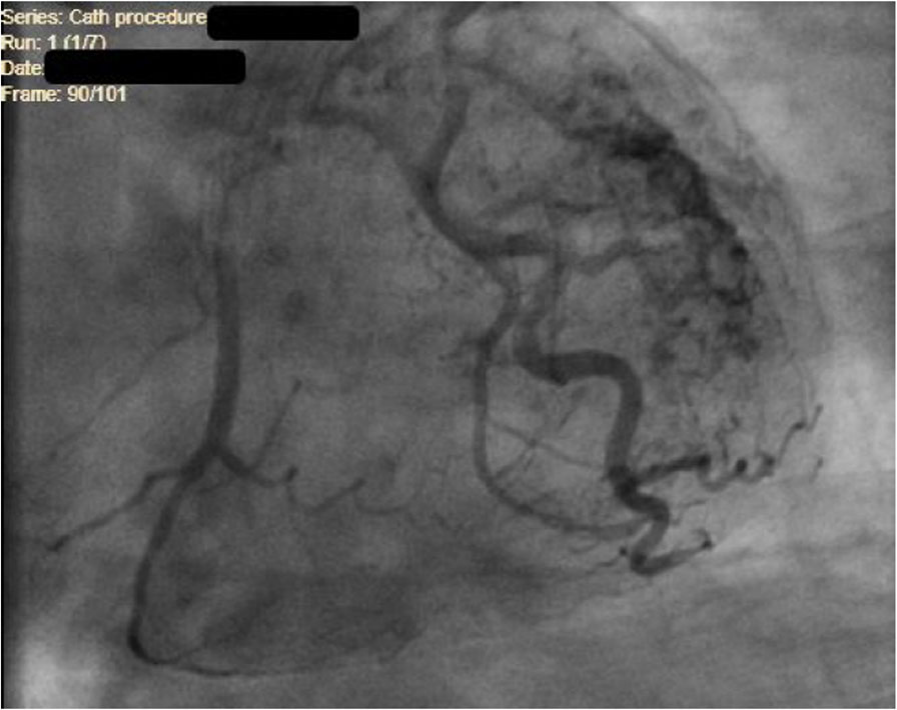
Coronary artery fistula forming a plexus at termination

The involved coronary artery is often dilated because of increased blood flow and can also exhibit a tortuous course depending on the volume of shunted blood. According to angiographic classification of Sakakibara *et al*. there are two categories of CAF, Type A, with proximal coronary dilation at the origin of fistula and normal distal end, Type B, coronary dilatation over the entire length [6, 7]. Morphologically, fistula could have a variable drainage at the termination, comprising of single or multiple communicating channels. It can also terminate as a maze of fine vessels forming a plexus with extensive mural distribution as seen in our case (Fig. 1).

Clinical symptoms may vary depending on site of origin, termination, and size; largely depending on the amount of blood flowing through the fistula. The main presenting symptom of the patient described in this case was chest pain due to ischemia. The proposed pathophysiologic mechanism is reduction in the intracoronary diastolic perfusion pressure in presence of numerous fistulas. Also, the myocardium beyond the origin of the multiple coronary fistulas suffers ischemia due to a coronary steal phenomenon manifesting as chest discomfort.

About half of the patients remain asymptomatic whereas the remaining half are often detected when an echocardiography or angiography is performed for an unrelated reason; however, cases of refractory heart failure, ischemia, and myocardial infarction have been reported. An EKG is usually nonspecific in the diagnosis of CAF, as seen in our case, although it can sometimes point to LV strain pattern or ischemic ST-T changes. The fistulae could be obstructed spontaneously as a result of atherosclerosis, leading to asymptomatic patients. In a case series of 51 patients with CAFs, angina pectoris occurred in 57% of cases in the absence of coronary atherosclerosis [8].

The goal of treatment is to occlude the fistula while providing normal coronary circulation. Surgical intervention includes the use of coils, detachable balloons, or alcohol injections. In our case, coronary artery-LV communications were too diffuse for surgical intervention and our patient was managed conservatively.

The management strategy for asymptomatic fistulas remains controversial. Asymptomatic fistulas should be managed invasively if the pulmonary to systemic flow ratio exceeds 1.5:1 or in presence of aneurysmal degeneration, which increases the risk of rupture. No guideline is established for management of patients with CAF; usually beta blockers, calcium channel blockers, or nitrates are recommended for ischemia management. Our patient was managed medically and so far has remained asymptomatic at 1-month and 6-month follow-up.

First described in Japan in 1991, the TCM is the syndrome of transient LV malfunction. The presentation, electrocardiogram and cardiac biomarker elevation may mimic acute myocardial infarction, and hence the condition is often misdiagnosed as ACS. But coronary angiography is frequently normal or with minimal abnormalities (<50% luminal obstruction). Regardless, the LV systolic function improves rapidly over a period of days to weeks.

**Fig. 2:**
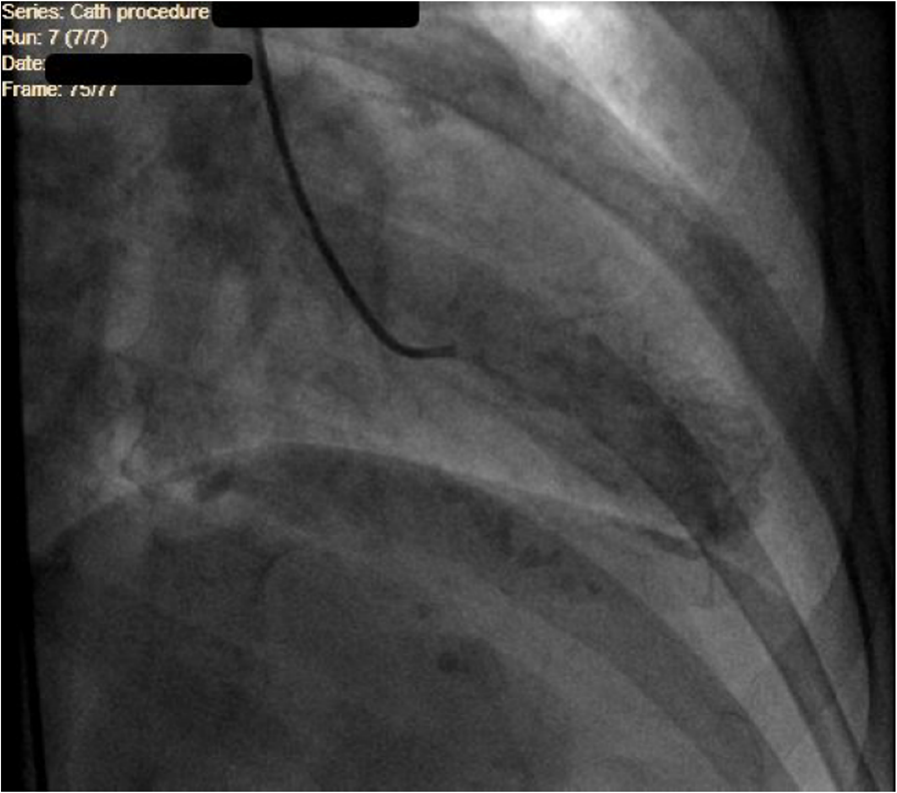
Apical ballooning of left ventricle on ventriculogram

Although the pathophysiology of TCM is not fully understood, severe pathophysiologic mechanisms have been proposed including multivessel coronary vasospasm and abnormalities in coronary microvascular function or catecholamine-mediated cardiotoxicity. The condition is usually triggered by acute medical illness or intense emotional or physical stress-induced adrenergic drive as seen in our patient. As explained by Lyon *et al*. [9], high levels of circulating epinephrine stimulate beta-2 adrenoceptor in ventricular myocytes, causing a switch from Gs to Gi protein. The Gi protein blocks the pro-apoptotic effect of intense beta-1 stimulation. In addition to that, the Gi protein is also negatively inotropic. The negative inotropic effect is greatest at the apex due to the maximum beta-adrenoceptor density. This hypothesis could be supported by the fact that several case reports describing pheochromocytoma-related cardiomyopathy demonstrated similar left ventricular wall motion abnormality [10]. Ninety percent of the reported cases comprised of postmenopausal women. One of the explanations proposes that sex hormones may stimulate sympathetic neurohormonal axis and coronary vasospasm [11]. Postmenopausal women tend to be more vulnerable to altered endothelial function due to reduced estrogen levels [12]. Clinical presentation is strikingly similar to that of an ACS with chest pain or dyspnea being most common, EKG usually shows ST elevation, T wave inversions, and pathological Q waves, moreover, cardiac biomarkers are frequently elevated, and TTE may show wall motion abnormality. However, coronary angiogram is completely normal or may show mild obstructive coronary pattern (<50% luminal narrowing), as seen in Fig. 1. Unlike ACS the disease is usually benign with full recovery in almost all reported cases so far. But unfortunately, the diagnosis can only be established after the coronary angiogram due to lack of clinical and radiologic characteristics to diagnose the condition with certainty. Our patient’s ventriculogram showed the classical apical ballooning of the left ventricle as seen in Fig. 2, with preserved function of the remaining myocardium consistent with TCM.

The treatment of acute cardiomyopathy depends on hemodynamic stability. If the patient is stable, it is not unreasonable to block excessive sympathetic tone by alpha and beta blockade, beta blockers are favored in dynamic LV obstruction, whereas alpha blockers are preferred in situations where there is outflow tract obstruction with severe hypotension. For hemodynamically unstable cases, intra-aortic balloon pump is modality of choice [13]. Due to the paucity of data, many questions regarding the chronic management strategies are unanswered. A retrospective study to look at the benefits of aspirin, ACE inhibitor, beta blocker, or calcium channel blocker did not show any percentage improvement in LV ejection fraction on admission, before discharge and 30 days post discharge [14]. More randomized controlled trials are needed to better guide the treatment strategies.

Our case represents an exceedingly rare association between left-sided CAF with TCM. We infer the pathophysiologic connection is the stress-induced overstimulation of myocardial beta-1 receptors, worsening the coronary steal in the setting of CAF, manifesting as ischemic pain, and also the stress-induced adrenergic drive causing the apical ballooning on ventriculogram that is characteristic of TCM

## Conclusions

Our case emphasizes the association between two discrete and rare entities: the coronary artery fistula and Takotsubo cardiomyopathy. Hence, it is very prudent that physicians should include this association in differential diagnosis when presented with postmenopausal women with a history of recent emotional or physical stress. Unfortunately, due to the lack of specific clinical, radiological, or laboratory characteristics, the coronary angiography remains the gold standard allowing the clinician to diagnose with certainty and to withhold unnecessary reperfusion therapy.

## Abbreviations

ACS: Acute coronary syndrome
BMI: Body mass index
CAF: Coronary artery fistula
CCF: Coronary cameral fistula
ECHO: Echocardiogram
ED: emergency department
EKG: Electrocardiogram
LAD: left anterior descending
LV: Left ventricle
TCM: Takotsubo cardiomyopathy
